# Rectal perforation following SpaceOAR placement combined with permanent prostate brachytherapy

**DOI:** 10.1002/iju5.12769

**Published:** 2024-08-13

**Authors:** Masashi Morita, Mayo Tanabe, Chisa Kinugawa, Saori Nakamura, Satoshi Amano, Kota Nishimura, Jin Yamatoya, Tetsuo Noguchi, Aya Hiramatsu, Takashi Fukagai

**Affiliations:** ^1^ Department of Urology Showa University Koto Toyosu Hospital Tokyo Japan; ^2^ Digestive Diseases Center Showa University Koto Toyosu Hospital Tokyo Japan; ^3^ Department of Urology Showa University School of Medicine Tokyo Japan

**Keywords:** prostate brachytherapy, rectal perforation, rectal ulcer, SpaceOAR

## Abstract

**Introduction:**

We report a case of rectal perforation following SpaceOAR placement utilized with iodine‐125 low‐dose‐rate brachytherapy for prostate cancer.

**Case presentation:**

A 65‐year‐old patient with localized prostate cancer underwent SpaceOAR placement following LDR‐BT. No significant issues occurred with the SpaceOAR procedure, and no abnormalities were found on the next day's T2‐weighted magnetic resonance imaging. Two weeks later, a colonoscopy was performed due to mucus stools revealing rectal perforation attributed to SpaceOAR. By maintaining Macrogol 4000 and a low residue diet, the perforation healed within 6 months.

**Conclusion:**

Rectal ulcers and perforations are the most common severe adverse events from SpaceOAR placement. Effective management strategies are crucial since complications can't be entirely avoided, even with skilled surgeons.

**Abbreviations & Acronyms**
AEs: adverse events
CS: colonoscopy
IMRT: intensity‐modulated radiation therapy
LDR‐BT: iodine‐125 low‐dose‐rate brachytherapy
RT: radiation therapy
RWI: rectal wall infiltration
SAbR: high‐dose stereotactic ablative radiation therapy
SOAR: SpaceOAR
T2‐MRI: T2‐weighted magnetic resonance imaging

**Keynote message**

Rectal ulcers and perforations are the most frequently encountered procedural complications associated with SOAR. Even deep ulcers or perforations may heal conservatively with early detection if no concurrent infection exists.

## Introduction

The SOAR system (Boston Scientific, Marlborough, MA, USA) significantly reduces ≧G2 rectal AEs when used with IMRT.^1^ It is also reported that 98.7% of physicians found the procedure easy despite their initial experience.^1^ Our previous report of SOAR with LDR‐BT also found that 94% of cases were successfully placed.^2^ While the procedure is safe and beneficial for rectal sparing, some cases required surgery due to procedure‐related AEs, and some intended RT could not be performed.^3^, ^4^ We report an early‐onset rectal perforation case after SOAR combined with LDR‐BT.

## Case presentation

**Fig. 1:**
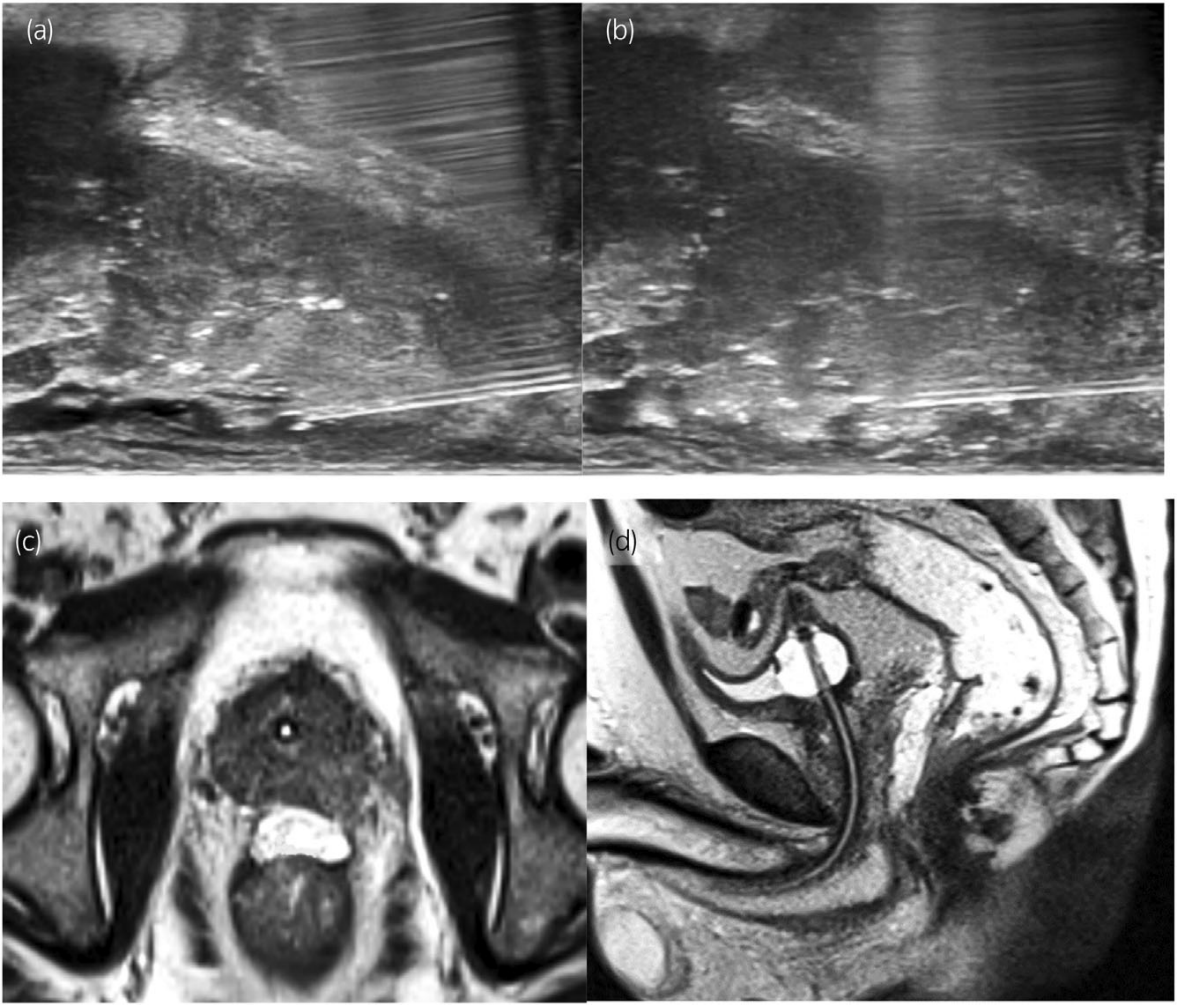
Ultrasound imaging during procedure (a, b) shows no abnormalities. T2‐MRI images from the following day (c, d) indicate successful gel placement.

**Fig. 2:**
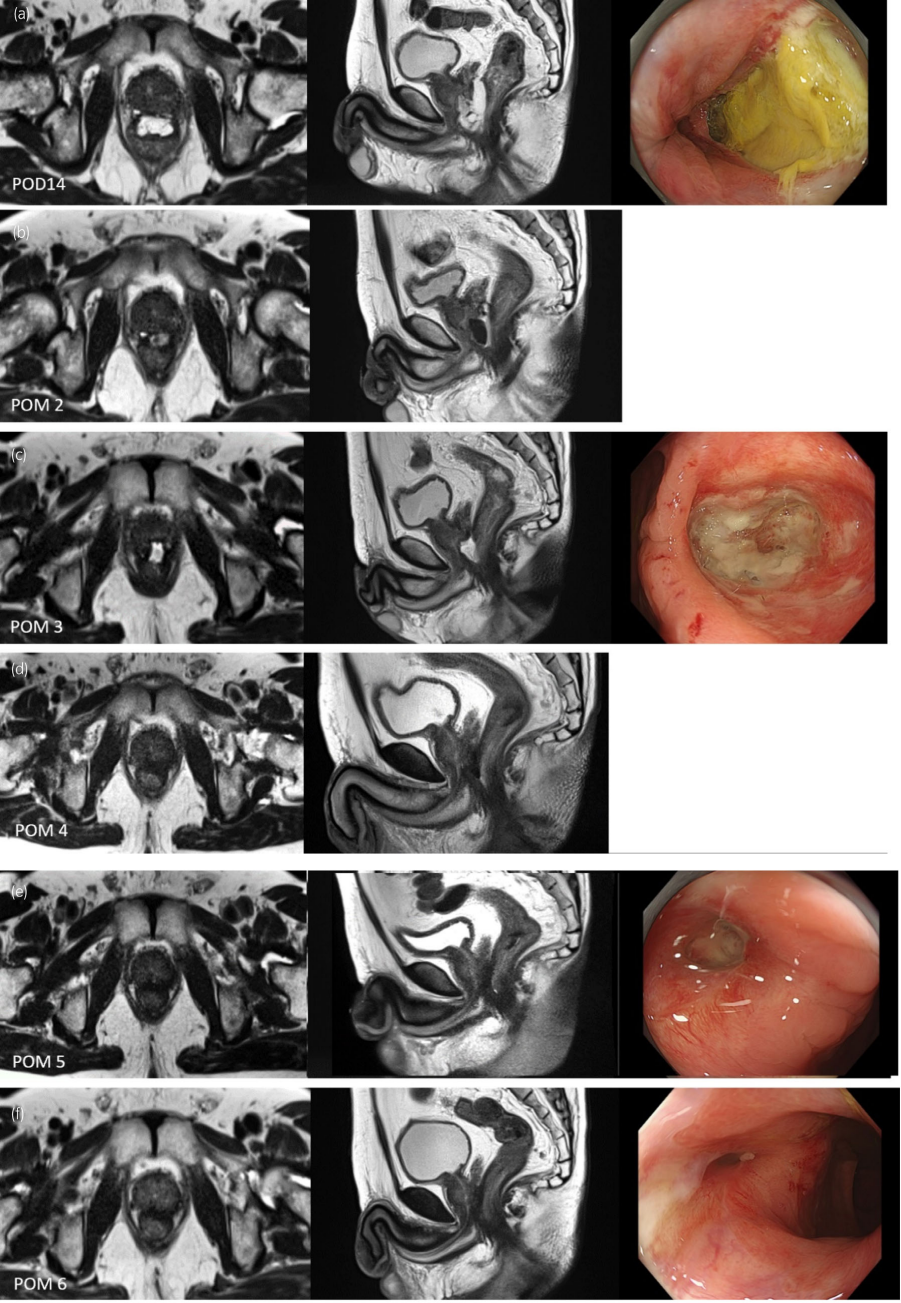
Temporal changes in T2‐MRI and CS findings.

A 65‐year‐old male with an initial prostate‐specific antigen of 6.80 ng/mL, Gleason score of 4 + 3, and cT2aN0M0 underwent LDR‐BT. The patient had no history of bowel disease. A physician with experience in 560 cases performed the SOAR placement following LDR‐BT. The procedure was done without particular issues (Fig. 1a,b). The T2‐MRI performed the following day also showed no abnormalities such as RWI (Fig. 1c,d). At a scheduled visit after 2 weeks, the patient reported mucus stools starting on the fourth postoperative day, with no fever or bloody stools. Due to early‐onset gastrointestinal symptoms, T2‐MRI was done. SOAR infiltration toward the prostate side was seen at the apex, with a slight suspicion of RWI (Fig. 2a), prompting CS. CS revealed extensive ulceration on the anterior wall of the rectum without surrounding vascular inflammation (Fig. 2a), leading to a diagnosis of SOAR‐induced ulceration. Multiple physicians reviewed the procedure video; however, there were no clear abnormal findings. A conservative approach was chosen, involving the administration of Macrogol 4000 and a low‐residue diet, with monthly follow‐up visits and MRI monitoring. Although symptoms worsened most in the first month, limited to mucus stools, there were no signs of inflammation like fever or pain, and blood tests remained normal. No unscheduled visits occurred due to symptom exacerbation. By the second month, SOAR absorption began, with air images suggestive of fistula on T2‐MRI (Fig. 2b). By the third month, seeds were visible beyond the rectal wall on CS (Fig. 2c), indicating complete recto‐prostatic fistula formation. Although hyperbaric oxygen therapy was recommended, the patient declined due to commuting from a distant location. Mucus stools were reduced in the fourth month. By the fifth month, there was marked improvement in the perforation site on MRI and CS (Fig. 2e). By the sixth month, the mucosal surface had almost fully recovered (Fig. 2f).

## Discussion

Despite its simplicity and safety, various SOAR‐related AEs have been reported. According to reports using The Manufacturer and User Facility Device Experience database,^3^, ^4^ device‐related AEs of ≥G2 appear most frequently associated with ulcers or perforations resulting from RWI. RWI cannot be entirely avoided solely through the operator's skill and proficiency. This is because the technique itself involves needle insertion beyond the rectal hump and into the Denonvilliers' thin layer, sometimes under unclear ultrasound imaging.

To the best of our knowledge, there have been seven case reports of rectal ulcers or perforations attributed to SOAR, including ours, up to the present (Table 1). It is anticipated that there will be many more unreported cases. We did not include cases where the onset occurred with symptoms such as abscess formation, and ulcers or perforations developed secondarily. The majority did not happen during the initial experience.^5^, ^6^, ^10^ The reason is unknown, but our experience was also seen in the 561st case during 617 SOAR procedures. In five reports, early T2‐MRI was performed after SOAR procedure to assess the placement. Among these, RWI was suspected in two reports,^9^, ^10^ although no findings were detected in the others. In our case as well, no abnormalities were detected on the T2‐MRI. We routinely perform T2‐MRI the day after SOAR. In two cases, we suspected RWI and performed CS the next day. CS showed no perforation, and the rectal mucosa was normal. Thus, both cases planned LDR‐BT plus IMRT, boosted IMRT performed as scheduled, with no significant rectal AEs. RWI without rectal mucosal abnormalities likely allows the safe continuation of planned RTs. Therefore, postoperative MRI is valuable for investigating the presence of RWI,^11^, ^12^ and if RWI is detected, performing CS to evaluate the rectal mucosal surface may provide valuable information for safely conducting planned RT. However, early postoperative T2‐MRI often does not reveal findings leading to fistula or ulcer formation. Detecting early signs of bowel symptoms, which are less likely with SOAR placement, is important. CS should be performed to check for ulcers or fistulas, and RT should be delayed until healing if these are present.

**Table 1:** Reports of rectal ulcer/perforation following SOAR placement

Reports	Modality	Abnormality during SOAR procedure	Early post‐SOAR MRI	Initial symptoms	Signs of infection	Time to discover complication	The modality used for diagnosis	Postponement or cancellation of RT	Treatment for ulceration or perforation	Progress/status
Klotz *et al*. (2013)^5^	IMRT	Nothing in particular	Performed	Unknown	None	At the completion of 38 Gy of RT	Unknown	Subsequent RT is postponed	Observation	Improved by POM3
Teh *et al*. (2014)^6^	LDR	Coagulation observed during two‐component injection	Not performed	Frequency, mucous stool, etc.	None	POM2	DRE, CS	N/A	Low fiber diet	Improved by POM3
Iinuma *et al*. (2019)^7^	LDR+EBRT	Nothing in particular	Not performed	Mucous and blood stool	None	POM1	MRI, CS	RT postponed until recovery, reduced dosage	Observation	Improved by POM2
Imai *et al*. (2020)^8^	IMRT	Multiple needle puncture	Performed	Frequency, perianal pain, decrease in hemoglobin	Perianal pain, afebrile	POM3	Enhanced CT, CS	IMRT proceeded as scheduled	Transfusion, intravenous fluids, and fasting (hospitalization)	Improved on the 5th day of hospitalization
Dinh *et al*. (2020)^9^	IMRT	Nothing in particular	Performed (RWI s/o)	Rectal urgency, mucous stool	Perirectal pain, afebrile	POM 2	CT, MRI, CS	IMRT proceeded as scheduled	Hyperbaric oxygen	Improved by POM5
McLaughlin *et al*. (2021)^10^	SAbR	Nothing in particular	Performed (RWI +)	Perineal fullness, hemorrhoids, etc.	None	POM 6	CS	SAbR proceeded as scheduled	Hyperbaric oxygen→	Cystectomy, colostomy
Present report (2024)	LDR	Nothing in particular	Performed	Mucous stool	None	POD 14	MRI, CS	N/A	Low fiber diet, enema	Improved by POM9

Furthermore, the problem is that RWI, which can cause perforation or ulcers, is often undetectable during surgery. One factor is the slight mixing of bubbles during gel injection, making the ultrasound image unclear. The case reports listed in Table 1 also indicate a lack of awareness of RWI during the procedure, although some reports mention minor issues encountered during treatment. Teh *et al*.^6^ experienced coagulation during the two‐component injection process, indicating the possibility of multiple needle punctures. Similarly, Imai *et al*.^8^ reported multiple needle punctures. It is most likely that the rectal hump, which is the narrowest part of the puncture route at the apex, is unconsciously being punctured by the needle. A pinhole might form, allowing SOAR to flow into the intestinal wall under pressure. Additionally, the mass effect from SOAR retention may cause localized ischemia, leading to larger perforations. Although our video check showed no abnormalities, the lowest needle often punctures the rectal hump during prior LDR‐BT, potentially allowing SOAR infiltration through these tiny holes.

## Conclusion

While rare, perforations and ulcers from SOAR can occur. Without infection, deep ulcers can heal conservatively. Early detection and proper management are crucial to prevent additional surgeries or abandonment of RT.

## Author contributions

Masashi Morita: Conceptualization; data curation; writing – original draft. Mayo Tanabe: Conceptualization. Chisa Kinugawa: Writing – review and editing. Saori Nakamura: Writing – review and editing. Satoshi Amano: Writing – review and editing. Kota Nishimura: Writing – review and editing. Jin Yamatoya: Writing – review and editing. Tetsuo Noguchi: Writing – review and editing. Aya Hiramatsu: Writing – review and editing. Takashi Fukagai: Writing – review and editing.

## Conflict of interest

The authors declare no conflict of interest.

## Approval of the research protocol by an Institutional Reviewer Board

This study has been approved by the ethics committee of our institution (CR2024001‐B).

## Informed consent

Not applicable.

## Registry and the Registration No. of the study/trial

Not applicable.

## Animal studies

Not applicable.
